# Defining the therapeutic corridor of stability in enzyme replacement therapy for Pompe disease: a position statement

**DOI:** 10.1186/s13023-026-04518-9

**Published:** 2026-07-28

**Authors:** Benedikt Schoser

**Affiliations:** Department of Neurology, Friedrich-Baur-Institute, LMU University Hospital, LMU Medizin, Ludwig-Maximilians-Universität München, München, Germany

**Keywords:** Pompe disease, Glycogen storage disease type II, Enzyme replacement therapy, Alglucosidase alfa, Avalglucosidase alfa, Cipaglucosidase alfa, Miglustat, Forced vital capacity, Six-minute walk test, Therapeutic corridor, Treatment switching, Real-world evidence, Biomarkers, ORPHA:365

## Abstract

**Background:**

Pompe disease, also known as glycogen storage disease type II, is a rare, progressive lysosomal storage disorder caused by pathogenic variants in the *GAA* gene. Enzyme replacement therapy has transformed the natural history of the disease; however, with three agents now approved — alglucosidase alfa, avalglucosidase alfa, and cipaglucosidase alfa plus miglustat — clinicians lack a consensus quantitative framework for determining when a patient has left the zone of therapeutic stability and requires treatment reassessment, escalation, or switching.

**Methods:**

A structured synthesis was conducted of Phase 2 and Phase 3 randomized controlled trials, open-label extension studies, registry analyses, real-world observational datasets, switching studies, scoping review evidence on global diagnostic and epidemiological variation, and consensus recommendations evaluating approved enzyme replacement therapies for Pompe disease, with an emphasis on late-onset Pompe disease. Outcome domains evaluated included forced vital capacity percentage predicted, six-minute walk test distance, urinary hexose tetrasaccharide, serum creatine kinase, maximal inspiratory and expiratory pressures, anti-drug antibody titers, functional motor scales, and patient-reported outcomes.

**Results:**

Across LOTS, COMET and its 97- and 145-week extensions, PROPEL and ATB200-07, ATB200-02, the STIG real-world cohort, the International Pompe Registry, European Pompe Consortium start–switch–stop criteria, post hoc PROPEL switching analyses, real-world alglucosidase-to-avalglucosidase switching data, and the first multicenter real-world analysis of switching to next-generation enzyme replacement therapies, a coherent therapeutic corridor of stability emerges. Forced vital capacity change within approximately − 1% to + 5% per year defines the respiratory stability corridor; a confirmed decline exceeding 5% over 12 months constitutes an alarm threshold. For the six-minute walk test, stabilization within approximately ± 25 m of the peak is an adequate response, whereas a confirmed decline of more than 25 m from the peak is actionable. Biomarker improvement or normalization of urinary hexose tetrasaccharide and creatine kinase is achievable with next-generation enzyme replacement therapies and serves as an early surrogate of enhanced lysosomal glycogen clearance. Multicenter real-world switching data indicate that transitions between enzyme replacement therapy preparations are generally feasible and associated with clinical stability in late-onset Pompe disease.

**Conclusions:**

This formal, multi-domain therapeutic corridor of stability for enzyme replacement therapy in Pompe disease is grounded in available Phase 2, Phase 3, extension, registry, consensus, and real-world evidence. The framework supports structured monitoring, timely reassessment of treatment, and personalized management for patients receiving long-term enzyme replacement therapy. Prospective validation with standardized monitoring is required.

**Clinical trial number:**

Not applicable.

## Background

Pompe disease (Online Mendelian Inheritance in Man #232300; Orphanet ORPHA:365) is an autosomal recessive lysosomal glycogen storage disorder caused by pathogenic variants in the *GAA* gene, resulting in deficiency of acid alpha-glucosidase (also known formerly as acid maltase). Progressive lysosomal glycogen accumulation in skeletal, cardiac, and smooth muscle leads to the hallmark clinical features of proximal limb-girdle and axial muscle weakness, respiratory failure, and, in the classic infantile form, hypertrophic cardiomyopathy [1]. The clinical spectrum extends from rapidly fatal classic infantile-onset Pompe disease, presenting in the first weeks or months of life, to a broad continuum of late-onset Pompe disease phenotypes presenting from childhood through late adulthood, till very late-onset beyond age 50.

The epidemiology of Pompe disease remains heterogeneous across regions because of differences in newborn screening implementation, diagnostic methods, case definitions, and access to confirmatory testing. A recent international scoping review by Giugliani and colleagues [2] highlighted substantial global variation in prevalence and incidence estimates and reinforced the need for harmonized diagnostic pathways, registry structures, and longitudinal outcome definitions. This heterogeneity has direct implications for treatment monitoring: a corridor framework must be sufficiently standardized to enable comparison across health systems while remaining flexible enough to accommodate regional differences in diagnosis, baseline severity, treatment access, and follow-up infrastructure.

Since the regulatory approval of alglucosidase alfa in Europe in 2006 and in the United States in 2010, enzyme replacement therapy has been the primary approved disease-modifying treatment for Pompe disease. Alglucosidase alfa dramatically improved survival in infantile-onset Pompe disease and stabilized motor and respiratory function in late-onset Pompe disease under pivotal trial conditions [3]. However, longitudinal real-world evidence has documented a well-characterized secondary decline in multiple functional domains after several years of alglucosidase alfa treatment, attributable in part to suboptimal cellular uptake via the cation-independent mannose-6-phosphate receptor and, in some patients, to immunogenicity with development of anti-recombinant human acid alpha-glucosidase antibodies [4, 5].

Two next-generation enzyme replacement therapies have now been approved. Avalglucosidase alfa incorporates synthetic high-affinity bis-mannose-6-phosphate glycans to enhance cation-independent mannose-6-phosphate receptor-mediated lysosomal enzyme targeting. Cipaglucosidase alfa plus miglustat combines a recombinant human acid alpha-glucosidase enriched for bis-mannose-6-phosphate content with miglustat, an iminosugar pharmacological chaperone that stabilizes the enzyme in circulation [6–8]. Both agents have demonstrated improved biomarker suppression and functional stabilization compared with alglucosidase alfa in clinical trial settings, and real-world data on switching from alglucosidase alfa to next-generation therapies are now emerging [9–11].

The advent of next-generation enzyme replacement therapies has shifted Pompe disease management from the binary question of whether to treat to the more complex question of whether a given patient is responding adequately to a specific treatment over time. Recent reviews of enzyme replacement therapy in adults with Pompe disease emphasize that early clinical trial and real-world data suggest potential advantages of next-generation agents, while acknowledging the lack of direct head-to-head comparisons between all approved therapies and the need for personalized assessment of outcomes [12]. Broader clinical insights from enzyme replacement therapy across metabolic storage disorders similarly show that improved survival reveals new long-term disease manifestations and unresolved questions regarding tissue targeting, treatment durability, and patient-centered long-term care [13].

The European Pompe Consortium EPOC updated its start, switch, and stop criteria in 2024 in response to the availability of next-generation therapies. These recommendations highlight the need for structured longitudinal assessment, including six-monthly monitoring of muscle function, consideration of magnetic resonance imaging of the muscles as supportive evidence, and explicit evaluation of whether patients should start, switch, or discontinue enzyme replacement therapy [14]. These recommendations provide a consensus foundation for clinical decision-making, but, by themselves, they do not define a quantitative, multi-domain therapeutic corridor of stability.

The recent multicentre real-world switching analysis by Mendelsohn et al. represents an important advance because it moves the evidence base beyond single-center or small observational switching series and provides multicentre evidence that transitions between enzyme replacement therapy preparations in late-onset Pompe disease are generally feasible and associated with clinical stability [10]. This finding is highly relevant to the proposed corridor concept: if treatment switching is feasible and can maintain stability, then clinicians require a structured framework to identify which patients should be monitored, reassessed, or switched, and when such a decision should be made.

Despite these advances, no widely adopted quantitative framework defines when a patient on enzyme replacement therapy has entered a therapeutically unacceptable zone of decline and requires treatment reassessment or switching. Clinicians must interpret complex, multimodal outcome trajectories in a disease characterized by small patient numbers, high inter-individual variability, limited direct head-to-head evidence between therapies, and substantial measurement variability in respiratory and functional outcomes. This Position Statement synthesizes the Phase 2, Phase 3, extension, registry, consensus, real-world, and epidemiological evidence base to propose a formal therapeutic corridor of stability for enzyme replacement therapy in Pompe disease across eight clinically relevant outcome domains.

## Evidence synthesis

### Overview of included evidence

The evidence base includes clinical trials, extension studies, registry analyses, real-world cohorts, switching studies, consensus recommendations, epidemiological scoping reviews, and management roadmaps. Studies and recommendations were selected based on relevance to treatment adequacy, longitudinal monitoring, functional stability, respiratory stability, biomarker response, switching feasibility, or patient-centered management (Table 1). This Position Statement is based on a narrative, expert synthesis of the published evidence rather than on a systematic review or a formal consensus process: no systematic database search, PRISMA-based study selection, formal grading of evidence (for example, GRADE), or risk-of-bias assessment was performed, and no external Delphi or consensus methodology was used. Accordingly, the corridor boundaries proposed below are author-proposed, evidence-informed thresholds derived from the author’s interpretation of trial, extension, registry, and real-world data, and are not validated decision rules or consensus guideline criteria. Where a threshold is supported directly by a specific dataset this is indicated in the text; where it reflects extrapolation or expert judgement it is labelled as author-proposed and requiring prospective validation.

**Table 1:** Summary of key evidence informing the therapeutic corridor

Study or source	Design	ERT/comparator or focus	*N*	Duration / follow-up	Primary relevance to corridor
LOTS, NCT00158600	Phase 3 RCT	Alglucosidase alfa vs. placebo	90	78 weeks	6MWT: +25.1 m vs. + 3.4 m; FVC: +1.4% vs. − 0.9% [3]
COMET, NCT02782741	Phase 3 RCT	Avalglucosidase alfa vs. alglucosidase alfa	100	49 weeks	FVC: +2.89% vs. + 0.46%; 6MWT: +32 m vs. + 2 m [6]
COMET OLE, 97 weeks	Phase 3 extension	Avalglucosidase alfa	95	97 weeks	FVC: +2.65% in continued avalglucosidase group; +0.36% after switch from alglucosidase alfa; 6MWT maintained [15]
COMET OLE, 145 weeks	Phase 3 extension	Avalglucosidase alfa	88	145 weeks	FVC: +1.38% in original avalglucosidase arm; +1.25% after crossover; functional stability sustained [4]
PROPEL, NCT03729362	Phase 3 RCT	Cipaglucosidase alfa plus miglustat vs. alglucosidase alfa plus placebo	123	52 weeks	6MWT % predicted: +4.1% vs. + 1.6%; FVC: −0.9% vs. − 4.0% [7]
ATB200-02	Phase 1/2 open-label	Cipaglucosidase alfa plus miglustat	20 initially; expanded cohort	≥ 18 months	FVC improved in ERT-naïve patients; CK and Hex4 reduced across cohorts; 6MWT improved [16]
ATB200-07 OLE	Phase 3 extension	Cipaglucosidase alfa plus miglustat	118	104 weeks	ERT-experienced patients maintained 6MWT and FVC; CK and Hex4 improved [17]
STIG, NCT02824068	Real-world retrospective cohort	Alglucosidase alfa	68	Mean 7.03 years; up to 10 years	Initial improvement followed by secondary decline; FVC − 14.93% at 10 years [5]
Carter et al. 2024	Real-world observational series	Switch from alglucosidase alfa to avalglucosidase alfa	15	Median 12 months	CK and Hex4 significantly reduced; 8 of 12 patients stable or improved in 6MWT [9]
Mendelsohn et al. 2025	Multicentre real-world switching analysis	Switching between ERT preparations, including next-generation ERTs	Multicentre LOPD cohort	Real-world follow-up	Switching was generally feasible and associated with clinical stability in LOPD; prospective standardised monitoring recommended [10]
Wenninger et al. (PROPEL post hoc) 2026	Post hoc effect size analysis	Switch from alglucosidase alfa to cipaglucosidase alfa plus miglustat	95 ERT-experienced participants	52 weeks	Switching to cipaglucosidase alfa plus miglustat associated with improvement or stability across most outcomes [11]
Wenninger et al. (PROPEL switching)	Phase 3 switching analysis	Cipaglucosidase alfa plus miglustat after alglucosidase alfa	PROPEL switch population	≥ 52 weeks	Clinically important improvements in 6MWT and FVC after switch [8]
International Pompe Registry / adult ERT review	Registry and review	ERTs in adults with Pompe disease	> 600 in registry context	Long-term	Earlier ERT initiation associated with better long-term FVC preservation; next-generation ERTs require personalised assessment [12]
EPOC triple-S criteria	European consensus	Start, switch, stop ERT	25 experts	2024 update	Supports structured start/switch/stop decision-making and six-monthly monitoring [14]

Abbreviations: 6MWT, six-minute walk test; CK, creatine kinase; EPOC, European Pompe Consortium; ERT, enzyme replacement therapy; FVC, forced vital capacity; Hex4, urinary hexose tetrasaccharide; LOPD, late-onset Pompe disease; OLE, open-label extension; RCT, randomized controlled trial

### Respiratory function: forced vital capacity

In untreated late-onset Pompe disease, meta-analytic data indicate progressive respiratory decline, with forced vital capacity percentage predicted to deteriorate over time [15]. This natural history provides an essential lower bound for the therapeutic corridor: any enzyme replacement therapy that maintains forced vital capacity above the expected rate of decline in the untreated state delivers incremental functional benefit.

The LOTS trial demonstrated a mean improvement in forced vital capacity of + 1.4% with alglucosidase alfa versus − 0.9% with placebo at 78 weeks [3]. The 10-year multinational STIG real-world study documented an initial improvement followed by a secondary sustained decline, with forced vital capacity percentage predicted to decrease by a mean of 14.93% from baseline over 10 years, despite continuous alglucosidase alfa [5]. This trajectory is critical because it defines a long-term negative reference boundary: patients following a STIG-like decline while on enzyme replacement therapy are moving below the desired therapeutic corridor.

In COMET, avalglucosidase alfa produced an LS mean forced vital capacity increase of + 2.89% versus + 0.46% with alglucosidase alfa at 49 weeks [6]. This advantage was maintained in the 97-week extension, with + 2.65% in the continued avalglucosidase group and + 0.36% after alglucosidase-to-avalglucosidase crossover [16]. At 145 weeks, forced vital capacity remained stable, with + 1.38% in the original avalglucosidase arm and + 1.25% in the crossover arm, supporting durable respiratory stabilization over nearly three years [4].

In PROPEL, cipaglucosidase alfa plus miglustat demonstrated a significant advantage for the key secondary respiratory endpoint, with a mean sitting forced vital capacity change of − 0.9% versus − 4.0% for alglucosidase alfa plus placebo [7]. In the ATB200-07 open-label extension, ERT-experienced patients maintained respiratory stability over 104 weeks [17]. A post hoc effect size analysis of PROPEL further supports the clinical relevance of switching in ERT-experienced patients: those remaining on alglucosidase alfa plus placebo generally showed worsening or stability across most outcomes, whereas those switched to cipaglucosidase alfa plus miglustat mostly showed improvement or stability, with no significant worsening across assessed outcomes [11]. A complementary PROPEL switching analysis confirmed clinically important improvements in six-minute walk distance and forced vital capacity after switching from alglucosidase alfa to cipaglucosidase alfa plus miglustat [8].

The real-world switching literature strengthens the clinical relevance of this corridor. Carter et al. demonstrated that switching from alglucosidase alfa to avalglucosidase alfa was associated with functional stability in most evaluable patients and significant biomarker improvement [9]. Mendelsohn et al. extended this evidence by reporting the first multicentre real-world analysis of switching to next-generation enzyme replacement therapies in late-onset Pompe disease, concluding that transitions between enzyme replacement therapy preparations are generally feasible and associated with clinical stability [10]. Together, these data support the concept that a confirmed decline in forced vital capacity exceeding approximately 5% over 12 months should trigger a structured reassessment and consideration of switching, particularly when accompanied by deterioration in motor outcomes, biomarkers, respiratory pressures, or patient-reported function.

### Motor function: six-minute walk test

The six-minute walk test integrates musculoskeletal endurance, cardiovascular performance, and respiratory reserve, and remains one of the most widely used measures of functional capacity in late-onset Pompe disease. A minimum clinically important difference of approximately 25–30 m is commonly used in neuromuscular disorders, and percentage predicted change is also used in Pompe-specific analyses. In this framework, “baseline” denotes the six-minute walk test distance recorded at initiation of the current enzyme replacement therapy or, when the corridor is used to evaluate a switching decision, the distance recorded immediately before the switch; “peak” denotes the maximal distance the individual patient achieves on the current therapy. The alarm threshold is therefore assessed as a confirmed loss from this individual peak, or as a relative loss exceeding 10% from the individual baseline, rather than as a fixed absolute distance applied uniformly across patients.

In LOTS, alglucosidase alfa produced a mean six-minute walk test improvement of + 25.1 m versus + 3.4 m for placebo [3]. In COMET, avalglucosidase alfa produced a mean improvement of approximately + 32 m compared with approximately + 2 m with alglucosidase alfa at 49 weeks [6]. This improvement was sustained through the 97- and 145-week extensions, with functional stability persisting after crossover from alglucosidase alfa to avalglucosidase alfa [4, 16].

In PROPEL, the six-minute walk test percentage was predicted to increase by 4.1% with cipaglucosidase alfa plus miglustat versus 1.6% with alglucosidase alfa plus placebo at 52 weeks [7]. At 104 weeks in the open-label extension, ERT-experienced patients maintained functional benefit [17]. The PROPEL post hoc switching analysis adds further support by showing that ERT-experienced patients switched from alglucosidase alfa to cipaglucosidase alfa plus miglustat demonstrated significant improvements in six-minute walk distance, both absolute and percentage predicted, while maintaining stability or improvement across most other outcomes [11], and a complementary PROPEL switching analysis reported clinically important improvements in six-minute walk distance after switching [8].

Real-world switching evidence provides clinically important context. Carter et al. reported that 8 of 12 evaluable patients were stable or improved in the six-minute walk test performance after switching from alglucosidase alfa to avalglucosidase alfa, with patients with mild-to-moderate baseline impairment generally faring better than those with more advanced disease [9]. Mendelsohn et al. further support the feasibility of ERT switching in routine multicentre late-onset Pompe disease practice, with clinical stability observed after transitions between enzyme replacement therapy preparations [10]. These data support a corridor in which a change in the six-minute walk test of approximately ± 25 m from peak is considered compatible with stability, while a confirmed decline greater than 25 m from peak should prompt treatment review.

### Biomarkers: urinary hexose tetrasaccharide and creatine kinase

Urinary hexose tetrasaccharide is a glycogen-derived biomarker of lysosomal glycogen burden and was first established as a useful measure of treatment response in Pompe disease nearly two decades ago [18]. Its interpretation as a monitoring biomarker is nonetheless subject to several preanalytical and analytical influences, including renal function, hydration status, age, diet, and differences in assay methodology and inter-laboratory standardisation, all of which should be considered when serial values are compared. Serum creatine kinase reflects ongoing skeletal muscle membrane damage and frequently improves with effective enzyme replacement therapy. However, a decline in creatine kinase must be interpreted with caution, because normalization does not invariably indicate treatment efficacy: in advanced disease, a falling creatine kinase may also accompany progressive loss of muscle mass and fibro-fatty muscle replacement rather than reduced ongoing muscle injury. Creatine kinase should therefore always be interpreted together with functional, respiratory, and, where available, muscle imaging measures. Although neither biomarker should be interpreted in isolation, both are useful adjuncts to respiratory and motor outcomes.

Both avalglucosidase alfa and cipaglucosidase alfa plus miglustat achieved greater reductions in disease-relevant biomarkers than alglucosidase alfa in pivotal and extension studies [6, 7, 17, 19]. In the real-world switching cohort by Carter et al., switching from alglucosidase alfa to avalglucosidase alfa was associated with statistically significant reductions in creatine kinase and urinary hexose tetrasaccharide, with biomarker normalization more frequent after switching [9]. The PROPEL post hoc effect size analysis also showed significant improvements in biomarkers among ERT-experienced patients switched to cipaglucosidase alfa plus miglustat [11].

The multicentre real-world switching analysis by Mendelsohn et al. further supports the role of structured multi-domain monitoring after switching to next-generation enzyme replacement therapies [10]. The conclusion that switching is generally feasible and associated with clinical stability is particularly relevant when biomarker improvement accompanies stabilization in forced vital capacity and six-minute walk test performance. Within the proposed corridor, biomarker normalization or sustained downward trends within 6–12 months after ERT initiation or switching should be regarded as favorable early signals, whereas persistent elevation or rising trends should prompt review of adherence, infusion adequacy, immunogenicity, and treatment effectiveness.

### Respiratory pressures, functional scales, and patient-reported outcomes

Maximal inspiratory and expiratory pressures provide complementary information to forced vital capacity, particularly in patients with diaphragmatic weakness, early nocturnal hypoventilation, or discordance between subjective respiratory symptoms and spirometric findings. Although these measures are less consistently reported across trials, stability within approximately ± 5% of the predicted value is consistent with treatment adequacy, whereas confirmed decline exceeding 10% should trigger review.

Functional motor scales, such as the Gait, Stairs, Gower, and Chair scales, the Medical Research Council sum score, timed tests, and patient-reported outcomes, such as the SF-36 physical component, provide important complementary information. Their value is particularly high when respiratory and six-minute walk test measures are difficult to interpret because of orthopedic comorbidity, fatigue, obesity, intercurrent infection, or variable effort. The PROPEL post hoc analysis is particularly relevant to this broader multidomain view because switching from alglucosidase alfa to cipaglucosidase alfa plus miglustat was associated not only with respiratory and walking outcomes but also with improvements in manual muscle testing, PROMIS-Fatigue, physician global impression, subject global impression, and biomarker measures [11].

Mendelsohn et al. reinforce the importance of systematic monitoring across multiple domains after switching between enzyme replacement therapy preparations [10]. These findings support an approach in which no single parameter should drive treatment switching in isolation unless the decline is severe, reproducible, and clinically meaningful. Rather, patients should be judged to have left the therapeutic corridor when deterioration is confirmed across two or more consecutive assessments, across two or more outcome domains, or when one major domain deteriorates in parallel with worsening biomarkers or patient-reported function.

The 2026 patient-centered management roadmap further supports this position by emphasizing longitudinal, multidisciplinary, patient-centered care in Pompe disease [20].

In practice, this means that the therapeutic corridor should be used not as a rigid algorithm but as a shared clinical framework linking objective measures, patient goals, daily function, fatigue, respiratory burden, treatment burden, and quality of life.

### Immunogenicity: anti-drug antibodies

Anti-recombinant human acid alpha-glucosidase antibodies are detectable in a significant proportion of patients receiving enzyme replacement therapy and may modulate therapeutic efficacy. Sustained high titers have been associated with reduced enzyme delivery, attenuated biomarker response, and clinical deterioration, particularly in high-risk infantile-onset Pompe disease. Much of the evidence linking very high sustained titers to loss of efficacy derives from high-risk infantile-onset Pompe disease and is extrapolated to the late-onset setting, where the relationship is weaker and more heterogeneous; late-onset-specific data are therefore emphasised here [21]. For the purposes of this framework, a “high” anti-drug antibody titer is taken pragmatically to mean a sustained titer at or above approximately 1:12,800 confirmed on repeat testing, consistent with thresholds commonly applied in the Pompe literature, while recognising that no universally validated cut-off exists and that neutralising activity, rather than titer alone, is often the more relevant determinant of clinical impact [21]. In late-onset Pompe disease, the relationship between anti-drug antibodies and outcome is more variable but remains clinically relevant when rising titers occur in parallel with functional decline or worsening biomarkers. A recent longitudinal analysis of 111 adults with Pompe disease reinforced this nuanced relationship: high, sustained anti-drug antibody titers were associated with an increased risk of infusion-associated reactions, and although titers did not significantly affect efficacy at the group level, neutralizing activity or high titers could negatively influence the disease course in individual patients [21].

Within the therapeutic corridor framework, anti-drug antibody monitoring is most informative when interpreted longitudinally and in context. Low, stable, or declining titers are compatible with continued stability. Sustained high or rising titers, especially when accompanied by declining forced vital capacity, worsening six-minute walk test performance, increasing urinary hexose tetrasaccharide, or increasing creatine kinase, should prompt a structured review of immunogenicity, infusion reactions, treatment adequacy, and possible therapeutic switching.

## Position statement: the therapeutic corridor of stability

Synthesizing the evidence summarized above, a therapeutic corridor of stability for patients with Pompe disease receiving enzyme replacement therapy can be proposed. This framework defines, for each outcome domain, a stability zone within which treatment is considered to be adequately controlling disease progression, an alarm threshold beyond which treatment review and potential change should be considered, and an optimal response benchmark representing best-case outcomes achievable with current next-generation enzyme replacement therapies.

The corridor is not intended to replace clinical judgment. Rather, it provides a reproducible structure for clinical monitoring, registry design, multidisciplinary review, patient-centered decision-making, and prospective validation. Single measurements should be interpreted cautiously, especially for effort-dependent outcomes such as the six-minute walk test and respiratory pressure testing. Treatment decisions should generally be based on confirmed trends, repeated assessments, and concordance across domains (Tables 2 and 3).

**Table 2:** FVC percentage predicted change from baseline across treatment arms at key timepoints

Timepoint	Natural history	Alglucosidase alfa	Avalglucosidase alfa	Cipaglucosidase alfa plus miglustat	Source
Baseline	0	0	0	0	—
Approximately 12 months	Decline expected	+ 1.4% in LOTS	+ 2.89% in COMET	−0.9% sitting FVC in PROPEL	[3, 6, 7]
Approximately 24 months	Continued decline expected	Long-term real-world stability variable	+ 2.65% at 97 weeks in continued avalglucosidase group	Respiratory stability maintained in ATB200-07	[15, 17]
Approximately 36 months	Progressive decline expected	Secondary decline observed in long-term real-world data	+ 1.38% at 145 weeks in original avalglucosidase arm; +1.25% after crossover	Longer-term data support stability in extension cohorts	[4, 5, 17]
Approximately 5 years	Progressive decline expected	Decline evident in long-term observational cohorts	Long-term real-world data still emerging	Long-term real-world data still emerging	[5, 10, 12]
Approximately 10 years	Severe cumulative decline expected	−14.93% in STIG real-world cohort	Not yet established	Not yet established	[5]

Abbreviations: FVC, forced vital capacity; LOTS, Late-Onset Treatment Study; PROPEL, Phase 3 cipaglucosidase alfa plus miglustat trial. Note: the values in this table are drawn from studies of differing design, population, and duration and are not directly or statistically comparable; in particular, the trial-derived changes over approximately 49–145 weeks and the STIG real-world estimate over 10 years reflect different cohorts, follow-up periods, and analytic methods and should not be read as head-to-head comparisons

**Table 3:** The therapeutic corridor of stability in Pompe disease enzyme replacement therapy: proposed thresholds by outcome domain

Outcome domain	Stability zone	Alarm threshold	Optimal response	Evidence base
FVC percentage predicted	Change approximately − 1% to + 5% per year	Confirmed decline > 5% over 12 months	Improvement > 2% or sustained long-term stability	Phase 3, extensions, registry, real-world switching [3–12, 15–17]
Six-minute walk test distance	Change approximately − 25 m to + 50 m from peak; approximately ± 5% predicted	Confirmed decline > 25 m from peak or > 10% from baseline	Improvement ≥ 30 m or sustained stability	Phase 3, extensions, post hoc switching, real-world switching [3–12, 15–17]
Urinary Hex4	Sustained reduction; near or within upper limit of normal	Persistent elevation above upper limit of normal or rising trend	Normalisation or major sustained reduction	Phase 1/2, Phase 3, post hoc, real-world switching [5–11, 16, 17, 19]
Serum creatine kinase	Sustained reduction; trending toward normal	Persistent > 3× upper limit of normal or rising trend	Normalisation or major sustained reduction	Phase 3, extensions, post hoc, real-world switching [5, 7, 9–11, 16, 17]
Maximal inspiratory/expiratory pressures	Stable within approximately ± 5% predicted	Confirmed decline > 10% from baseline	Improvement > 5% or sustained stability	Phase 3 and clinical monitoring evidence [4, 6, 7, 14, 15]
Anti-drug antibody titre	Negative, low, stable, or declining	Sustained high or rising titre in context of clinical or biomarker decline	Negative, low, or clinically non-significant titre	Phase 1/2, Phase 3, immunogenicity evidence [14, 16, 17]
Functional motor scales	Stable within approximately ± 1–2 points, depending on scale	Sustained worsening > 2 points or clinically meaningful functional loss	Improvement ≥ 2 points or preserved independence	Real-world, post hoc, and clinical monitoring evidence [9–11, 14, 20]
Patient-reported outcomes	Stable within approximately ± 5 points on validated scales	Decline > 10 points or sustained deterioration in physical function	Improvement > 10 points or sustained quality-of-life stability	Phase 3, post hoc, and patient-centred monitoring evidence [6, 7, 11, 14, 15, 20]

Abbreviations: FVC, forced vital capacity; Hex4, urinary hexose tetrasaccharide

Values represent evidence-informed guidance derived from published data and expert synthesis. Formal validation in prospective cohorts is required. The stability zones, alarm thresholds, and optimal-response benchmarks in this table are author-proposed and, except where a specific supporting study is cited in the corresponding text, have not been prospectively validated; the reference block listed for each domain indicates the evidence that informed that domain overall rather than a validation of the individual numerical cut-off. These cut-offs are therefore intended as a structure for prospective testing, not as established decision rules

## Discussion

This Position Statement proposes a structured therapeutic corridor of stability for enzyme replacement therapy in Pompe disease. The framework is grounded in a synthesis of Phase 2, Phase 3, extension, registry, consensus, real-world, switching, and epidemiological evidence across approved enzyme replacement therapies. It is intended to help clinicians distinguish acceptable stability from clinically meaningful deterioration and to support timely, consistent, evidence-based, and patient-centered treatment reassessment.

Five central observations support the corridor framework:

First, the evidence base is now sufficiently mature to define quantitative stability thresholds. LOTS demonstrated the initial efficacy of alglucosidase alfa in late-onset Pompe disease [3]. COMET and its extensions demonstrated sustained respiratory and functional stability with avalglucosidase alfa, including after crossover from alglucosidase alfa [4, 6, 16]. PROPEL and ATB200 extension data demonstrated respiratory and biomarker advantages for cipaglucosidase alfa plus miglustat over alglucosidase alfa in relevant domains [7, 17, 19]. These trial and extension datasets provide the controlled evidence foundation for the upper and central corridor boundaries.

Second, long-term real-world data define the lower boundary of the corridor. The STIG study showed that even under continuous alglucosidase alfa treatment, a subset of patients experiences secondary decline over long-term follow-up, including substantial loss of forced vital capacity over 10 years [5]. This finding is central to the corridor concept because it demonstrates that “being on ERT” is not synonymous with adequate control. Instead, treatment adequacy must be judged by longitudinal stability across respiratory, motor, biomarker, immunological, functional, and patient-reported domains.

Third, switching evidence now supports the feasibility of acting on corridor-defined decline. Carter et al. showed that switching from alglucosidase alfa to avalglucosidase alfa was associated with improvements in biomarkers and functional stability in many patients [9]. Mendelsohn et al. extended this evidence through the first multicentre real-world analysis of switching to next-generation enzyme replacement therapies in late-onset Pompe disease, concluding that transitions between enzyme replacement therapy preparations are generally feasible and associated with clinical stability [10]. The PROPEL post hoc effect size analysis further supports this concept by showing that ERT-experienced patients switched from alglucosidase alfa to cipaglucosidase alfa plus miglustat achieved improvement or stability across most outcomes, whereas those remaining on alglucosidase alfa plus placebo more often showed stability or worsening [11]. Complementary PROPEL switching analyses confirmed clinically important improvements in six-minute walk distance and forced vital capacity after switching [8]. Together, these data provide pragmatic support for the clinical actionability of the corridor. These switching data must, however, be interpreted with caution at the point of use: Carter et al. and Mendelsohn et al. are retrospective, non-randomised, clinician-driven cohorts with small sample sizes and no non-switching comparator, so indication bias, regression to the mean, and the preferential switching of patients already perceived to be declining may all inflate the apparent benefit of switching. The corridor should therefore treat these data as supportive rather than confirmatory when a switching threshold is applied.

Fourth, consensus recommendations now explicitly recognize the importance of structured decision-making for start, switch, and stop decisions. The 2024 European Pompe Consortium triple-S criteria provide a consensus framework for initiating, switching, and discontinuing enzyme replacement therapy and recommend long-term monitoring of muscle function every 6 months [14]. The therapeutic corridor proposed here complements these recommendations by translating longitudinal monitoring into quantitative stability zones and alarm thresholds.

Fifth, Pompe disease management is increasingly patient-centered and longitudinal. The 2026 patient-centered roadmap emphasizes the need for multidisciplinary care, shared decision-making, and sustained attention to patient priorities, treatment burden, fatigue, respiratory symptoms, and quality of life [20]. The therapeutic corridor is therefore not only a numerical framework but also a practical communication tool for clinicians and patients to discuss whether treatment is achieving the goal that matters most: maintaining meaningful function over time. A further consideration is whether stability is always a sufficient therapeutic goal. The corridor is framed around the detection of decline, but some of the evidence synthesised here—including the PROPEL post hoc analyses and the real-world series of Carter et al.—indicates that a proportion of patients who appeared stable on their prior therapy nonetheless improved after switching to a next-generation enzyme replacement therapy. This raises the question of whether continued stability should always be accepted when a superior response might be achievable with another agent. At the same time, switching a clinically stable patient carries costs, treatment burden, uncertainty, and the risk of losing an established response. The corridor does not resolve this tension; rather, remaining within the stability zone should prompt a shared, individualised discussion of whether to maintain current therapy or to trial an alternative, weighing the potential for additional gain against the burden and risk of change.

The proposed forced vital capacity threshold for confirmed decline, exceeding 5% over 12 months, reflects both clinical plausibility and alignment with observed trial and real-world trajectories. Smaller annual fluctuations may reflect test variability, respiratory infection, effort, posture, or intercurrent illness. By contrast, reproducible decline exceeding 5%, particularly when accompanied by deterioration in six-minute walk test performance, biomarkers, respiratory pressures, or patient-reported function, is unlikely to represent noise alone and should trigger multidisciplinary treatment review.

The proposed six-minute walk test threshold of confirmed decline greater than 25 m from peak is similarly grounded in clinically meaningful change. Because the six-minute walk test is effort-dependent and influenced by orthopedic, cardiopulmonary, motivational, and environmental factors, single measurements should not be overinterpreted. However, repeated decline exceeding approximately 25 m, especially when coupled with worsening respiratory or biomarker data, should be considered actionable.

The integration of biomarkers strengthens the corridor framework. Urinary Hex4 and serum creatine kinase are not replacements for clinical outcome measures, but they provide early and objective signals of glycogen burden and muscle injury [18]. Declining or normalizing biomarkers after initiation or switching of enzyme replacement therapy support the biological treatment effect. Conversely, rising biomarkers may precede overt functional decline and should prompt reassessment of disease activity, adherence, infusion timing, immunogenicity, and therapeutic adequacy.

Recent therapeutic reviews also place the corridor in a broader context. The review of enzyme replacement therapies in adults with Pompe disease emphasizes that next-generation treatments may offer advantages over alglucosidase alfa, while acknowledging that the field still lacks direct head-to-head comparisons between all three approved therapies and requires more data to guide personalized start, stop, and switch decisions [12]. A broader review of enzyme replacement therapy in metabolic storage disorders further highlights that improved survival has revealed new long-term manifestations and unmet needs, including tissue targeting, treatment durability, and CNS accessibility for some disorders [13]. These observations support the need for monitoring frameworks that evolve as therapeutic goals change.

The epidemiological literature also has implications for the corridor. Global variation in diagnostic methods, newborn screening programs, and prevalence estimates means that patients may enter treatment pathways at different ages, disease stages, and baseline functional states [2]. Corridor thresholds must therefore be interpreted in light of diagnostic timing and baseline disease burden. Early-diagnosed patients may warrant tighter thresholds because preventable decline is more actionable, whereas patients with advanced, irreversible muscle replacement may require goals focused on slowing deterioration, preserving independence, and stabilizing respiratory burden. To make the corridor usable across the full severity spectrum, thresholds should be anchored to each patient’s own baseline and residual capacity rather than to population-level values. For ambulatory patients with preserved respiratory function, the quantitative thresholds proposed here apply directly. For more severely affected patients, the emphasis shifts from absolute thresholds to preservation of residual function. In non-ambulatory or wheelchair-dependent patients the six-minute walk test is not evaluable, and monitoring should instead rely on upper-limb and axial motor scales, timed function tests where feasible, respiratory parameters, biomarkers, and patient-reported outcomes. In patients requiring continuous or nocturnal ventilatory support, forced vital capacity is best tracked as an individualized relative change from the patient’s own recent baseline, supplemented by maximal inspiratory and expiratory pressures and by measures of ventilator dependency, with the treatment goal defined as stabilization of residual respiratory function and prevention of further decline. In both groups, a confirmed downward trend from the individual baseline in any evaluable domain—rather than the crossing of a fixed population threshold—should trigger structured reassessment.

Several limitations must be acknowledged. No direct head-to-head trial comparing avalglucosidase alfa with cipaglucosidase alfa plus miglustat exists, so the corridor cannot establish a hierarchy between next-generation enzyme replacement therapies. Many proposed thresholds are derived from late-onset adult cohorts and should not be automatically transferred to infantile-onset Pompe disease or pediatric late-onset populations without further validation. Real-world switching studies remain limited by small sample sizes, heterogeneous follow-up, potential selection bias, and incomplete standardization of outcome assessments. Some newer references are reviews, consensus documents, post hoc analyses, or scoping reviews rather than primary prospective trials; these sources are useful for framing the corridor but cannot substitute for prospective validation. Finally, the proposed thresholds represent author-proposed, evidence-informed boundaries rather than validated decision rules. In addition, baseline function strongly conditions the interpretation of change: patients with high baseline forced vital capacity or six-minute walk distance have limited room for measurable improvement, particularly over the short follow-up of the pivotal trials, so an apparent plateau in such patients may reflect a ceiling effect rather than inadequate treatment. Conversely, the aggregate mean changes reported for each therapy obscure substantial inter-individual heterogeneity—illustrated by the wide range of trajectories in the STIG cohort—driven by baseline severity, respiratory involvement, disease duration, and other patient-level factors; this heterogeneity limits the transferability of group-level thresholds to individuals and underscores the need for predictive biomarkers to identify likely responders and patients at risk of suboptimal response.

The next step is prospective validation. A dedicated multicentre study should evaluate whether patients identified as outside the corridor — for example, by confirmed forced vital capacity decline greater than 5% over 12 months, six-minute walk test decline greater than 25 m, worsening biomarkers, or multidomain deterioration — regain or maintain stability after switching to a next-generation enzyme replacement therapy. The primary validation endpoint could be re-entry into the therapeutic corridor within 12–24 months. Secondary endpoints should include respiratory slope, trajectory of the six-minute walk test, changes in Hex4 and creatine kinase, anti-drug antibody status, patient-reported physical function, treatment burden, safety, and treatment persistence.

As gene therapy, substrate reduction, improved enzyme targeting, newborn screening, and other disease-modifying strategies advance, the therapeutic corridor will need to expand. Future therapies may produce different patterns of response, including delayed stabilization, biomarker-first response, discordance between biochemical correction and functional recovery, or tissue-specific benefit. A corridor-based framework is well-suited to this evolving landscape because it focuses not on a single drug class but on the clinically meaningful question of whether the patient remains stable, improves, or continues to decline.

## Conclusions

This Position Statement defines a proposed therapeutic corridor of stability for enzyme replacement therapy in Pompe disease, grounded in Phase 2, Phase 3, extension, registry, consensus, real-world, and switching evidence across approved therapies. The corridor identifies stability zones, alarm thresholds, and optimal response benchmarks across respiratory, motor, biomarker, immunological, functional, and patient-reported domains.

The following position recommendations are proposed:

1. A confirmed forced vital capacity percentage predicted decline exceeding 5% over 12 months should be considered an alarm threshold requiring structured clinical review.
2. A confirmed decline in the six-minute walk test of more than 25 m from peak or greater than 10% from baseline should prompt reassessment of treatment adequacy.
3. Urinary Hex4 and serum creatine kinase should be monitored longitudinally as early biological indicators of glycogen burden and muscle injury. Normalization or sustained reduction supports treatment effectiveness, whereas persistent elevation or rising trends should trigger review.
4. Anti-drug antibody titers should be interpreted in context. Rising or sustained high titers accompanied by clinical or biomarker decline should prompt evaluation of immunogenicity and treatment effectiveness.
5. No single measurement, in isolation, should usually trigger treatment switching. Patients should be considered outside the therapeutic corridor when decline is confirmed across repeated assessments, across two or more domains, or when one major domain deteriorates in parallel with worsening biomarkers or patient-reported outcomes.
6. Real-world switching evidence, including the multicentre analysis by Mendelsohn et al. and post hoc PROPEL switching analyses, supports the feasibility of transitions between enzyme replacement therapy preparations and provides practical justification for structured reassessment and timely switching in selected patients [8, 10, 11].
7. The European Pompe Consortium start–switch–stop criteria, including the monitoring recommendation, should be used alongside the therapeutic corridor as a complementary consensus framework for structured treatment decisions [14].
8. Baseline disease severity remains a dominant determinant of long-term trajectory. Corridor thresholds must therefore be contextualized to individual baseline status, ambulatory capacity, respiratory reserve, comorbidities, treatment history, diagnostic timing, treatment burden, and patient goals.
9. Patient-centered outcomes, including fatigue, dyspnoea, daily function, treatment burden, and quality of life, should be incorporated into corridor-based monitoring, while recognising that validated patient-reported outcome data specific to enzyme replacement therapy switching in late-onset Pompe disease remain limited and warrant dedicated prospective collection.
10. The therapeutic corridor requires prospective validation using standardized monitoring and iterative refinement as longer-term data and new therapeutic modalities emerge.

Adoption of this framework by clinical teams, rare disease registries, health technology assessment bodies, and patient advocacy organizations may support more consistent, equitable, and timely treatment decision-making for people living with Pompe disease on long-term enzyme replacement therapy.

## Data Availability

No datasets were generated or analysed during the current study.

## Abbreviations

6MWT: Six-minute walk test
ADA: Anti-drug antibody
bis-M6P: Bis-mannose-6-phosphate
CI-MPR: Cation-independent mannose-6-phosphate receptor
CK: Creatine kinase
CRIM: Cross-reactive immunological material
EPOC: European Pompe Consortium
ERT: Enzyme replacement therapy
FVC: Forced vital capacity
GAA: Acid alpha-glucosidase
GSGC: Gait, Stairs, Gower, Chair scale
Hex4: Urinary hexose tetrasaccharide
IOPD: Infantile-onset Pompe disease
LOPD: Late-onset Pompe disease
MCID: Minimum clinically important difference
MEP: Maximal expiratory pressure
MIP: Maximal inspiratory pressure
MRC: Medical Research Council
OLE: Open-label extension
ORPHA: Orphanet identifier
PROMIS: Patient-Reported Outcomes Measurement Information System
RCT: Randomised controlled trial
rhGAA: Recombinant human acid alpha-glucosidase
ULN: Upper limit of normal
